# Unmasking the presentation of AKD in COVID-19

**DOI:** 10.1590/2175-8239-JBN-2025-E010en

**Published:** 2025-08-18

**Authors:** Mauricio Younes-Ibrahim

**Affiliations:** 1 Universidade do Estado do Rio de Janeiro, Rio de Janeiro, RJ, Brazil.; 2 Pontifícia Universidade Católica do Rio de Janeiro, Rio de Janeiro, RJ, Brazil.

The COVID-19 pandemic has had multiple impacts on renal health, with both short- and long-term effects manifesting in a variety of phenotypes. To understand this new disease and the uniqueness of its manifestations, studies are compiling clinical and pathophysiological information to correlate these findings with stablished renal syndromes.

Since the beginning of this century, the establishment of KDIGO (Kidney Disease Improving Global Outcomes) has significantly advanced nephrology practice worldwide by standardized definitions, classifications and clinical guidelines for kidney diseases. In 2020, due to difficulty in categorizing acute kidney disorders that did not fit the traditional syndromic criteria created for Acute Kidney Injury (AKI) and/or Chronic Kidney Disease (CKD), KDIGO introduced the term Acute Kidney Disease (AKD)^1^. AKD may share pathophysiological mechanisms with AKI or CKD, but it is distinguished by the timing of its identification. AKD has the potential to reveal early alterations in renal function and/or structure, thereby creating a critical window for therapeutic interventions to mitigate insults and prevent progression to overt AKI or CKD^2^.

AKI emerged as the predominant nephrological phenotype associated with COVID-19 worldwide^3^. Although the kidney is not the primary target of SARS-CoV2, viral tropism for the kidney, systemic effects of infection, and detection of AKD may serve as an early indicator of impending AKI, a morbidity associated with increased mortality and frequent need of renal replacement therapy. In the context of long COVID-19, AKD may also signal a risk of progression to CKD.

The syndromic diagnosis of AKI involves glomerulocentric criteria based on diuresis and reduced glomerular filtration rate (GFR). Modern tubular biomarkers are not yet widely available for clinical practice^4^. The identification of AKD is relevant in cases where, after a renal insult, there is a slow decline in GFR with a drop that does not meet the diagnostic AKI criteria. Likewise, AKD can serve as an early indicator of CKD triggered after an acute injury event, before the conventional 3-months timeframe required for its diagnosis^5^. In these contexts, the use of function and/or injury biomarkers helps in AKD diagnosis^6^.

The prospective observational multicenter study “Acute kidney disease in patients with Covid-19” by Lombardi et al.^7^, published in the current JBN issue, brings together clinical observations of renal involvement in patients with COVID-19 from 5 countries in Latin America. This series includes 360 hospitalized patients and found that approximately half had proteinuria and 76% had AKD. AKI, the most severe phenotype, occurred in 36.4%, 70% of whom developed AKI during hospitalization. The vast majority of cases was non-oliguric, and almost half was classified as KDIGO stage 3. AKI was considered community-onset when elevation of creatinine occurred in the first 24 hours. Regarding renal outcomes, half of the patients showed either total or partial recovery of kidney function.

Although creatinine values ​​at hospital admission were within the normal range, the cohort primarily consisted of elderly individuals, predominantly males in their sixties, with a significant presence of comorbidities such as arterial hypertension, diabetes, and obesity, which are recognized as independent causes of proteinuria and nephropathies. A possible hypothesis proposed in this study is that AKD, revealed by proteinuria in COVID-19 patients, is an early stage of AKI. However, this is unlikely to be a universal pattern, since 4% of patients developed AKI without proteinuria. The data corroborate the evidence that multiple possible mechanisms may contribute to the development of AKI in patients hospitalized with COVID-19. It is also noteworthy that not all individuals exhibit kidney involvement, and approximately 25% of the patients hospitalized in this series did not present kidney disease during hospitalization. Focusing on nephrological manifestations, the intensity of kidney impairment was directly related to clinical severity. The best prognosis was seen in patients without renal manifestations, and multivariate regression showed that mortality risk increased with the presence of proteinuria (4.4%) and with AKI (6.7%), and increased even further when AKI was associated with proteinuria (20.7%). Paradoxically, the risk of death was higher in patients with AKI KDIGO 2 than KDIGO 3. In agreement with other series, the severity of illness in this cohort was reflected by the high number of ICU admissions, invasive ventilatory support, mortality, and prolonged hospital stay. Finally, while vaccination did not significantly reduce AKI cases, it had a significant protective effect on proteinuria and was associated with improved clinical outcomes of patients hospitalized with COVID-19.

Pathological proteinuria may result from: 1- disturbances in glomerular permeability; 2- decreased tubular reabsorption (mediated by megalin); or 3- presence of abnormal proteins in the circulation. Experimental models reveal that tubular injuries are capable of causing acute proteinuria that precedes the reduction in GFR^8^. In this Latin American series of COVID-19, simple methods, such as urinalysis and dipstick testing, were used to identify proteinuria. Only qualitative data were computed and showed that both proteinuria, alone or associated with AKI, correlated with increased mortality. The literature on the presence of proteinuria associated with AKI in COVID-19 is vast^9^. In fact, glomerulopathies and Fanconi-like syndrome, with proteinuria associated with glycosuria, have been linked to more severe disease phenotypes^10^, showing that all segments of the nephron are potential targets for infection. More recently, experimental models have revealed that the SARS-CoV2 spike protein can downregulate both megalin expression and albumin endocytosis in renal tubular cells^11^.

Therefore, although the mechanisms involved have not been properly understood and previous comorbidities may have influenced the results of the cohort, the study contributes by showing that simple proteinuria may serve as an early biomarker of renal infectious involvement that is easily obtainable, capable of identifying AKD and has prognostic value in patients with COVID-19.
