# Supplementary material for: Unmasking the presentation of AKD in COVID-19
Source: J Bras Nefrol. 2025 Aug 18;47(3):e2025E010. doi: 10.1590/2175-8239-JBN-2025-E010en (PMC12360247; doi:10.1590/2175-8239-JBN-2025-E010en)
Supplement: Translation

# Translation: Desvendando a apresentação da DRA na COVID-19

**DOI:** 10.1590/2175-8239-JBN-2025-E010pt

**Authors:** Mauricio Younes-Ibrahim

**Affiliations:** 1 Universidade do Estado do Rio de Janeiro, Rio de Janeiro, RJ, Brasil.; 2 Pontifícia Universidade Católica do Rio de Janeiro, Rio de Janeiro, RJ, Brasil.

A pandemia de COVID-19 produziu múltiplos impactos na saúde renal dos pacientes, detectados a curto e a longo prazo, manifestados por meio de diferentes fenótipos. Para compreender essa nova doença e o ineditismo das observações, estudos com informações clínicas e fisiopatológicas têm buscado elencar seus achados nas síndromes renais já conhecidas.

Desde o início deste século, a criação do KDIGO (*Kidney Disease: Improving Global Outcomes*) tem beneficiado a prática da nefrologia mundial com sua iniciativa exitosa de padronizar definições, classificações e diretrizes clínicas para as doenças renais. Mais recentemente, em 2020, diante da dificuldade em reunir distúrbios renais agudos não alcançados pelos critérios sindrômicos criados para Injúria Renal Aguda (IRA) e/ou Doença Renal Crônica (DRC), o KDIGO introduziu o termo Doença Renal Aguda (DRA)^1^. As causas de DRA podem ter as mesmas origens fisiopatológicas da IRA ou da DRC, diferenciando-se pelo momento clínico em que são identificadas na linha do tempo. A DRA seria potencialmente capaz de revelar estágios iniciais de alterações na função e/ou estrutura renal, possibilitando, assim, a detecção de oportunidades para intervenções terapêuticas que mitiguem os insultos e retardem o desenvolvimento de IRA ou DRC^2^.

A IRA foi o principal fenótipo nefrológico exibido mundialmente pela COVID-19^3^. Embora o rim não seja um órgão primariamente acometido pelo SARS-CoV2, o tropismo viral para este órgão, os efeitos sistêmicos da infecção e a detecção de DRA podem prenunciar a IRA, morbidade que aumenta a mortalidade e frequentemente requer métodos de substituição da função renal. Nos diferentes aspectos de cronicidade na COVID-19 longa, a DRA também pode servir como alerta sobre a progressão para DRC.

O diagnóstico sindrômico de IRA utiliza critérios glomerulocêntricos, baseados na diurese e na redução da taxa de filtração glomerular. Biomarcadores tubulares modernos ainda não se encontram amplamente disponíveis para a prática clínica^4^. A identificação da DRA torna-se relevante quando, após um insulto renal, ocorre um lento declínio da Taxa de Filtração Glomerular (TFG), com uma queda que não seria suficiente para preencher os critérios de IRA. Do mesmo modo, a DRA pode identificar a progressão para DRC deflagrada após um evento de injúria aguda, antes dos 3 meses convencionados para o seu diagnóstico^5^. Nesses contextos, o uso de biomarcadores de função e/ou de lesão auxilia no diagnóstico da DRA^6^.

O estudo prospectivo, observacional e multicêntrico *“Acute kidney disease in patients with Covid-19. A prospective, multicenter, multinational study in Latin America”*, de Lombardi et al.^7^, publicado neste volume, reúne observações clínicas sobre o envolvimento renal em pacientes com COVID-19 de 5 diferentes países da América Latina. Esta série inclui 360 pacientes hospitalizados e identificou que cerca da metade cursou com proteinúria e 76% com DRA. A IRA, fenótipo mais grave, ocorreu em 36,4% dos casos, 70% delas intra-hospitalar, a grande maioria não-oligúrica e quase metade no estágio KDIGO 3. A IRA foi considerada comunitária quando a elevação da creatinina ocorreu nas primeiras 24h. Quanto ao desfecho renal, metade dos pacientes apresentou recuperação total ou parcial da função renal.

No conjunto dos dados, embora os valores de creatinina na admissão hospitalar estivessem dentro da faixa de normalidade, a coorte é composta por indivíduos idosos, com mediana de sexagenários do sexo masculino, e com expressiva presença de comorbidades como hipertensão arterial, diabetes e obesidade reconhecidas causas independentes de proteinúria e nefropatias. Uma possível hipótese levantada neste estudo é que a DRA, revelada pela proteinúria na COVID-19, represente um estágio inicial da IRA. Se assim ocorre, certamente não se trata de uma regra, já que 4% dos pacientes desenvolveram IRA sem proteinúria. Os dados corroboram as evidências de que há múltiplos mecanismos possíveis determinantes de IRA em pacientes hospitalizados com COVID-19. É relevante observar fenótipos da infecção que poupam os rins, sendo que cerca de 25% dos pacientes hospitalizados nesta série não apresentaram doença renal durante a internação. Com foco nas manifestações nefrológicas, a intensidade do comprometimento renal esteve diretamente relacionada à gravidade clínica. O melhor prognóstico foi observado na ausência de manifestação renal, e a regressão multivariada mostrou que o risco de mortalidade aumenta com a presença de proteinúria (4,4%), eleva-se com a IRA (6,7%) e piora quando a IRA está associada à proteinúria (20,7%). Paradoxalmente, o risco de óbito foi maior em pacientes com IRA KDIGO 2 do que naqueles no estágio KDIGO 3. Em concordância com outras séries, observou-se a gravidade da doença traduzida pelo elevado número de internações em UTI, necessidade de suporte ventilatório invasivo, mortalidade e tempo de permanência hospitalar. Por fim, a vacinação, embora não tenha demonstrado redução nos casos de IRA, exerceu um efeito protetor significativo sobre a proteinúria e o desfecho dos pacientes internados com COVID-19.

A proteinúria patológica pode resultar de: 1) distúrbios na permeabilidade glomerular; 2) diminuição da reabsorção tubular (mediada por megalina); ou 3) presença de proteínas anormais na circulação. Modelos experimentais revelam que injúrias tubulares são capazes de causar proteinúria aguda que precedem a redução da TFG^8^. Nesta série latino-americana de COVID-19, métodos simples como a urinálise e o teste com fita foram utilizados para identificar a presença de proteinúria. Apenas dados qualitativos foram computados e mostraram que ambas as formas de proteinúria, isolada ou associada à IRA, se correlacionaram com o aumento da mortalidade. A literatura sobre a presença de proteinúria associada à IRA na COVID-19 é extensa^9^. Inclusive, glomerulopatias e a Síndrome Fanconi-like, com proteinúria associada à glicosúria, conferiram fenótipos de gravidade à doença^10^, mostrando que todos os segmentos do néfron são potenciais alvos para a infecção. Mais recentemente, modelos experimentais revelaram que a proteína *spike* do SARS-CoV2 promove a redução tanto da expressão de megalina quanto da endocitose de albumina nas células tubulares renais^11^.

Portanto, embora os mecanismos envolvidos ainda não tenham sido devidamente compreendidos, e as comorbidades prévias possam ter influenciado os resultados da coorte, o estudo contribui ao evidenciar que a simples proteinúria parece se traduzir em um biomarcador precoce preditivo deste envolvimento infeccioso renal, sendo de fácil obtenção, capaz de identificar a DRA e com valor prognóstico em pacientes com COVID-19.
